# Insertion of Dye-Sensitized Solar Cells in Textiles using a Conventional Weaving Process

**DOI:** 10.1038/srep11022

**Published:** 2015-06-18

**Authors:** Min Ju Yun, Seung I. Cha, Seon Hee Seo, Han Seong kim, Dong Y. Lee

**Affiliations:** 1 Nano Hybrid Technology Research Center, Creative and Fundamental Research Division,Korea Electrotechnology Research Institute; 2 Department of Organic Material Science and Engineering, Pusan National University

## Abstract

Increasing demands for wearable energy sources and highly flexible, lightweight photovoltaic devices have stimulated the development of textile-structured solar cells. However, the former approach of wire-type solar cell fabrication, followed by weaving of these devices, has had limited success, due to device failure caused by high friction forces and tension forces during the weaving process. To overcome this limitation, we present a new approach for textile solar cell fabrication, in which dye-sensitized solar cell (DSSC) electrodes are incorporated into the textile during the weaving process, using the textile warp as a spacer to maintain the DSSC structure. Porous, dye-loaded TiO_2_-coated holed metal ribbon and Pt nanoparticle-loaded carbon yarn were used as the photoanode and counterelectrode, respectively. The highly flexible textile-based solar cell was fabricated using a common weaving process with a loom. The inserted DSSCs in the textile demonstrated an energy conversion efficiency of 2.63% (at 1 sun, 1.5 A.M.). Our results revealed that additional performance enhancement was possible by considering other electrode materials and textile structures, as well as where and how the DSSC electrodes are inserted. In addition, we demonstrated that the inserted DSSCs could be electrically connected using a parallel configuration.

Textile-related technologies, including weaving, printing, and dyeing, have been developed and modified throughout human history over the last millennium. These processes are so close to our everyday life that we sometimes fail to recognize their importance; however, it has been impossible to replace them with other technologies. In this respect, wearable devices have received much attention, with a focus on adopting the properties and forms of textiles. For energy-source wearable devices, the textile solar cell is considered to be a promising future technology^1^,^2^,^3^. Additionally, the characteristic performance of textiles, based on having a lightweight, mechanical robustness, and high flexibility, can facilitate the possible application of solar-cell panels to building-integrated solar cell (BIPV) or living-room interiors.

Considering such possible application fields, the textile solar cell properties should also include high electric power output under weak illumination, an esthetically pleasing appearance, and a low production cost. Dye-sensitized solar cells (DSSCs)^4^,^5^,^6^ meet all of these requirements. Thus, considerable research has been dedicated to developing textile-structured DSSCs over the last decade. In particular, metal-based meshes are highly bendable and electrically conductive but the sandwich-type cell structure is a fundamental problem for highly bendable DSSCs. It is vulnerable to electrical shorts during bending, and the strain is concentrated on the sealing gaskets or spacers during deformation. So we proposed the core-integrated structure before^7^,^8^.

And most textile DSSC studies to date have been based on wire-shaped^9^,^10^,^11^,^12^ or wire-integrated^13^,^14^ solar cells that can be easily woven into the textile. These studies focused mostly on the development of wire-shape DSSCs utilizing anodized Ti wires, TiO_2_ layer-deposited metal wires^15^,^16^,^17^,^18^, or optical fiber^9^,^10^,^11^,^12^,^13^,^14^,^15^,^16^,^17^,^18^, without consideration of the actual weaving process.

In weaving or other textile processes, such as braiding and knitting, yarn (an assembly of many filaments or wires) is generally used. Yarn is strong under frictional and tension loads, such as those encountered in industrial, high-speed weaving processes. However, in terms of manufacturing textile DSSCs, these conditions may destroy the photovoltaic surface layers, seriously degrading device performance. Thus, new fabrication techniques are required for textile DSSCs to accommodate the textile form and photovoltaic performance.

In this report, we describe a new fabrication process for textile-based DSSCs. Here, the DSSC electrode components, the photoanodes and counterelectrodes, were woven into the textile in the form of weft or wraps. The woven DSSC textile was then sewn onto common cloth, such as silk, cotton, and paper. Thus, the resulting textile solar cell consisted of a simple layered assembly of textile electrodes (not interlocked) as one sheet of textile or cloth^19^. Through this approach, the textile-based DSSC can be fabricated by means of weaving processes to form solar cell-inserted textiles. Additionally, we demonstrated that the inserted DSSCs could be electrically connected in parallel, similar to conventional DSSC modules prepared using transparent conductive oxide (TCO)-coated glass substrates.

Figure 1 shows the photoanode and counterelectrodes incorporated as the weft during the weaving process. Specifically, dye-loaded, porous TiO_2_-coated stainless steel (SUS304) ribbons with periodic holes were used for the photoanode; carbon yarn coated with Pt nanoparticles was used as the counterelectrode. The inserted DSSC components were surrounded by a conventional textile of glass yarn, as shown in Fig. 1(a) (see Supporting Information Fig. S1 for the detailed structure of the photoanode metal ribbon and counterelectrode carbon yarn); the inserted structures are shown more clearly in the cross-sectional view (Fig. 1(b)). Here, nylon filaments in the warp direction support the photoanode metal ribbon and maintain the spacing between the photoanode and counterelectrode to prevent an electrical short, until filled by the electrolyte. Additionally, the counterelectrode carbon yarns were supported by other nylon warps to maintain the textile structure, as shown in Figs 1(b,c). It is noteworthy that the geometry of the inserted electrodes (as weft) was determined by the relative stiffness of the nylon warp and electrode weft. Due to the high stiffness of the metal ribbon (SUS 304) and carbon yarn, the inserted electrode wefts maintained a straight shape, as shown in Fig. 1(c).

The procedure used to insert the DSSC electrodes into the textile during the weaving process is described in more detail in Fig. 2(a). Before insertion of the DSSCs, the conventional textiles were woven within the loom. To insert the DSSCs into the woven textile, first, the Pt nanoparticle-loaded carbon yarn was inserted as the weft between the photoanode-supporting nylon wire warp and carbon-yarn supporting nylon wires. After this process, the shed was closed, and the SUS ribbon for the photoanode was inserted as the weft above the carbon yarn. The conventional weaving process then continued. Through this process, the DSSCs can be inserted into conventional textiles, using warps that are originally located for weaving of conventional textiles.

The inserted DSSCs demonstrated an energy conversion efficiency of 2.63% under 1-sun illumination, as shown Fig. 2(b) (short-circuit current density: 5.78 mA cm^−2^, open-circuit voltage: 0.725 V; fill factor: 0.63). Additionally, the electrode components; *i.e*., the metal ribbon and carbon yarn, were sufficiently flexible to prevent bending of the surrounding textiles. Thus, the prepared DSSC textile was highly flexible, as shown in Fig. 2(c), with the fabricated textile bent under a radius of curvature of 1 cm. It should also be noted that the assembly processes described were performed using a six-heddle loom; thus, this method could be expanded to machine looms with proper modification for high-speed processing.

The energy conversion performance of the inserted DSSCs in the textile depends on the status of the electrodes before insertion and the structural factors presented by the textile structure. The inserted DSSC in the textile has two main components: the photoanode metal ribbon and the counterelectrode carbon yarn. For the photoanode electrode, consisting of a dye-loaded, porous TiO_2_ nanoparticle film and holed stainless steel (SUS304) ribbon, the thickness of the TiO_2_ layer can have a marked effect on the energy conversion performance. In particular, the structure of the photoanode, in which the electron collector (SUS304 ribbon) is located on the backside of the illuminated surface of the TiO_2_ layer (back-contacted structure), is different from the conventional sandwich-structured DSSCs, in which the illuminated TiO_2_ surface is attached to the electron collector (transparent conductive oxide layer). In this conventional back-contact configuration, the electrons generated near the TiO_2_ surface where the light intensity is strongest, travel a long distance to the collector, and hence have a higher probability of recombination. Therefore, the optimum thickness of the TiO_2_ layer that maximizes photon absorption and minimizes electron recombination should be determined.

To exclude other effects in the determination of the optimum TiO_2_ thickness, a model DSSC cell was prepared. The model DSSC consisted of a dye-loaded TiO_2_ layer deposited on a holed SUS 304 ribbon for the photoanode, and a conventional counterelectrode with Pt nanoparticles deposited on a fluorine-doped tin oxide (FTO)-coated glass substrate; the electrodes were separated by a paper spacer in place of the electrolyte. Because the DSSC in the textile has wire warps that separate the photoanode and counterelectrode to prevent an electrical short, a paper spacer was used to provide a closer representation of the textile DSSC for the optimization procedure. The thickness of the TiO_2_ layer was controlled by repeating the screen-printing process, resulting in layer thicknesses that ranged from 1.7 to 8 μm, as shown in Fig. 3(a). The energy conversion efficiency as a function of the TiO_2_ layer thickness is shown in Fig. 3(b). As the TiO_2_ layer thickness increased from 1.7 to 4.7 μm, the energy conversion efficiency increased from 1.35 to 2.2%. However, when the thickness of the TiO_2_ layer increased to 8 μm, the energy conversion efficiency dropped to 1.70%; additionally, a decrease was observed in the open-circuit voltage (V_oc_). Taken together, these results imply that the reduction in the energy conversion efficiency for the 8-μm TiO_2_ layer thickness was caused by electron recombination in the TiO_2_ layer. The effect of recombination on efficiency degradation was also evident in electrochemical impedance spectroscopy (EIS) results under open-circuit conditions (Fig. 3(c)). Here, the reaction peak near 10 Hz was associated with the recombination in the photoanode.

In addition to the photoanode, the size and distribution of the Pt nanoparticles in the carbon yarn was examined to determine their effect on DSSC performance. The Pt nanoparticle size and distribution depended on the Pt precursor (H_2_PtCl_6_∙6H_2_O) concentration in the dipping solution (Fig. 3(d)). For a precursor concentration of 10 mM, the Pt nanoparticles partially coated the carbon filament. As the concentration increased, the Pt-loaded coating became more homogeneous. However, for precursor concentrations >40 mM, the Pt nanoparticles created a Pt film that coated the carbon filament. The effect of these morphological changes on the performance of the inserted DSSC is shown in Fig. 3(e); the well-dispersed Pt in the carbon yarn provided a high current density and fill factor confirmed by low impedance in counter electrode as shown in Fig. 3(f). The optimum precursor concentration (20 mM) resulted in well-dispersed Pt in the carbon yarn and high DSSC energy conversion performance (see Supporting Information Fig. S2(a) for a Nyquist plot for Fig. 1(c) and Fig. S2(b) for a Bode plot of Fig. 3(f)).

The diameter of the nylon wires used to support the TiO_2_-coated stainless steel ribbon or Pt-deposited carbon fiber filaments is another critical factor that may improve the performance of the inserted DSSCs in the textile. The diameter of the nylon wire supporting the stainless steel ribbon may affect the distance between the photoanode and the counterelectrode, as well as the tension force among the nylon wires that are arranged as warps (Fig. 3(a)).

Another important consideration is the spacing between the photoanode metal ribbon and the carbon yarn counterelectrode for the textile DSSC. The spacing can be controlled by the thickness of the two warp wires: the photoanode-supporting warp nylon wire and the counterelectrode-supporting warp nylon wire, as shown in Fig. 4(a). An increase in the thickness of the photoanode-supporting warp increased the spacing between the electrodes, while the counterelectrode supporting warp provided a hill-shaped curved carbon yarn toward the photoanode, as shown in Fig. 4(a). As a result, although the photoanode-supporting warp was 190 μm in diameter, the spacing between the photoanode and the summit of the carbon yarn was only 75 μm. Additionally, if the photoanode-supporting warp had a diameter <150 μm, then the inserted DSSC experienced an electrical short during bending. Thus, photoanode-supporting wires with a diameter over 220 μm degraded the energy conversion efficiency of the inserted DSSCs, as shown in Fig. 4(b), by increasing the spacing between the electrodes; this result was confirmed by EIS results (Fig. 4(c)) that showed an increase in the impedance (Fig. 4(d)). Additionally, an increase in the diameter of the counter electrode-supporting warp straightened the carbon yarn and decreased the height of the hills on the warp. As a result, the spacing between the electrodes increased and the performance decreased, as shown in Fig. 4(b). The EIS results shown in Fig. 4(c) indicate that the inserted DSSCs with carbon yarn, supported by 300-μm-diameter wire warp, had higher impedance (approaching 10^3^–10^4^ Hz), compared with that of a 220-μm-diameter wire warp. Therefore, to minimize degradation, the warp diameter should be reduced to the point that the DSSCs are no longer electrically shorted (see Supporting Information Fig. S3 for detailed current density–voltage (*J*–*V*) curves and Bode plots). The inserted DSSCs in textile exhibited significant bending ability, the photovoltaic performance was measure by wrapping the cell around the rod of each radius of curvature. The photovoltaic performance was maintained at 85% of the flat cell value with bending at a 3 cm radius of curvature. (Fig. S4)

In practical applications, the inserted DSSC in the textile may need to be electrically connected with other DSSCs in the same textile or other devices, such as batteries or sensors. To show the possibility of electrical connection of DSSCs inserted in the textile, three DSSCs were electrically connected within the textile in parallel (Figs 5(a,b)), using Ti warp in the textile. The energy conversion performance, shown in Fig. 5(c), confirmed the parallel connection. Compared with the illumination of a single DSSC, three DSSCs connected in parallel produced a higher short-circuit current (2.5-fold increase) and a slightly lower open-circuit voltage. The decrease in the open-circuit voltage was caused by a characteristic effect of parallel circuits; i.e., the total voltage is determined by the lowest voltage. Thus, the inserted DSSCs in the textile can be connected within the textile with conductive wires or yarn to other devices or DSSCs to optimize their application or performance.

In summary, a new approach was proposed for incorporating DSSCs into textiles, in which the DSSC electrodes were incorporated during the weaving process and the textile warps were used as spacers to maintain the DSSC structure. Porous, dye-loaded TiO_2_-coated holed metal ribbon and Pt nanoparticle-loaded carbon yarn as the photoanode and counterelectrode, respectively, were used to create a highly flexible textile-based solar cell via a common weaving process using a loom. The inserted DSSCs in the textile demonstrated an energy conversion efficiency of 2.63% under 1 sun, 1.5 A.M. illumination, with the possibility of improvement by varying the electrode materials and textile structures, as well as where and how the DSSC electrodes are inserted. Thus, our approach provides new possibilities for wearable devices, especially solar cells, for future solar-energy applications.

## Experimental Details

An etched mesh of 304 stainless steel (Tech-Etch MicroEtch^®^) was used as a substrate for the photoanode. Etched mesh of 304 stainless steel has the regular arranged 76.2 μm holes and 23% opening. The thickness of etched mesh is 50.8 μm. Carbon fiber filaments (Hyosung^®^) were used as a counterelectrode. Transparent nylon wires (FRITZ^®^) were used as warps for core-integrated structure solar cells. For the frame part of the textile, glass fiber (D450 1/2, Hyunmin Fiber) was used as warps and wefts. The handy loom produced by Daesung High-tech was utilized for weaving.

The etched mesh was rinsed with acetone, ethanol, and deionized water by sonication and dried with nitrogen gas. After cleaning, the etched mesh was heat-treated at 480 °C for 1 h in air for oxidation of the metal surface. After oxidation, TiO_2_ paste, containing 20-nm TiO_2_ nanoparticles (EnB Korea) was deposited on the etched mesh by screen-printing (200 mesh, stainless steel), followed by heat treatment at 480 °C for 1 h in air. The active area of the photoanode was 8 × 3 mm^2^.

The carbon fiber filaments were rinsed and heat-treated using the method described for the etched mesh to remove impurities from the carbon fiber filament surface. Pt particles were deposited on the surface of the carbon fiber filaments by dipping the filaments in an aqueous solution of 20 mM H_2_PtCl_6_∙6H_2_O (Sigma Aldrich), followed by heat treatment at 400 °C for 30 min in air.

After fabricating and cleaning the photoanode and counterelectrode, the electrodes were woven into the textile as the weft. The inserted woven cell was then immersed in 0.3 mM ethanol solution of N719 dye (Solaronix) at room temperature for 20 h. After loading dye, the inserted DSSCs in textile was sealed with PET-based laminated pouch film (0.1 mm thick, Sindoh Commerce) using a commercial hot-roll-coating machine (Sindoh Commerce, TL-4600). Before sealing, a micro size hole was made on one side of the pouching film for acetonitrile-based electrolyte (Solaronix SA, AS50) filling using a syringe. An acetonitrile-based electrolyte (Solaronix, Iodolyte-AN50) was used as the electrolyte.

To confirm the effect of the thickness of the TiO_2_ layer, a counterelectrode was prepared by dropping an aqueous solution of 20 mM H_2_PtCl_6_∙6H_2_O onto FTO glass and masking the glass using 3M tape. The size of the counterelectrode was 10 × 5 mm^2^. The counterelectrode then underwent heat treatment at 400 °C for 30 min in air. To prevent an electrical short between the photoanode and counterelectrode and to retain the electrolyte, Hanji (a Korean traditional paper) was used as a spacer. To interconnect the three inserted woven DSSCs in parallel, 100-μm-diameter Ti wire (iNexus) was used as the warp. Field-emission scanning electron microscopy (FE-SEM, Hitachi S4800) was used to examine the sample surfaces. The energy conversion performance of the DSSCs was evaluated using a solar simulator (Abet Technologies, model Sun 2000, 1000 W Xe source, Keithley 2400 source meter) under 1.5-AM 1-sun conditions, calibrated by a KG-3 filter and NREL-certified reference cell without a mask. The impedance spectra were acquired under open-circuit, 1-sun conditions.

## Additional Information

**How to cite this article**: Ju Yun, M. *et al*. Insertion of Dye-Sensitized Solar Cells in Textiles using a Conventional Weaving Process. *Sci. Rep*. **5**, 11022; doi: 10.1038/srep11022 (2015).

## Supplementary Material

Supplementary Information

## Figures and Tables

**Figure 1:**
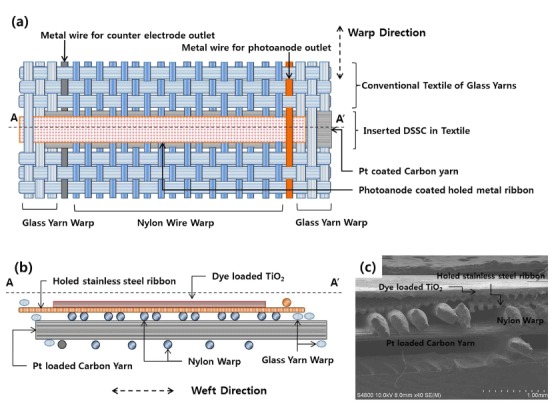
(**a**) Schematic illustration of the structure of the inserted dye-sensitized solar cells (DSSCs) in the textile in planar view and (**b**) cross-sectional view of the AA′ section shown in (**a**). (**c**) Scanning electron microscopy (SEM) images of the AA′ section in (**a**), showing the cross-sectional view of the inserted DSSC in the textile.

**Figure 2:**
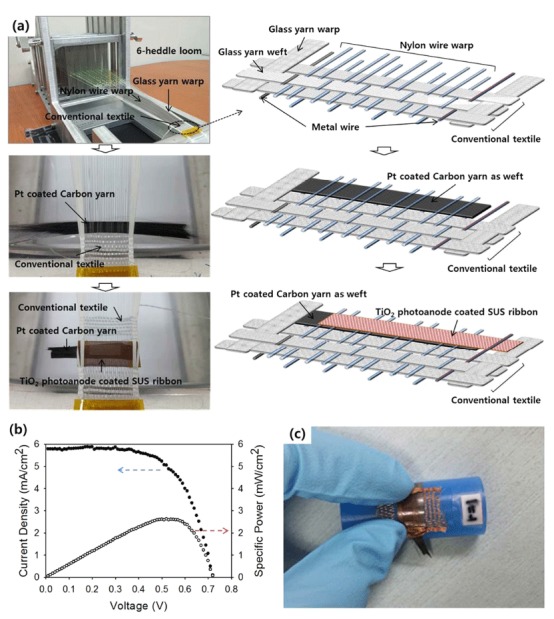
(**a**) Schematic illustration (right column) and photographs (left column) of the process of inserting DSSCs in the textile during the weaving process with a six-heddle loom. (**b**) Current density–voltage (*J*–*V*) characteristics and power–voltage characteristics of representative inserted DSSCs in the textile. (**c**) Photograph of an inserted DSSC textile bent on the surface of a rod with a 1-cm radius of curvature.

**Figure 3:**
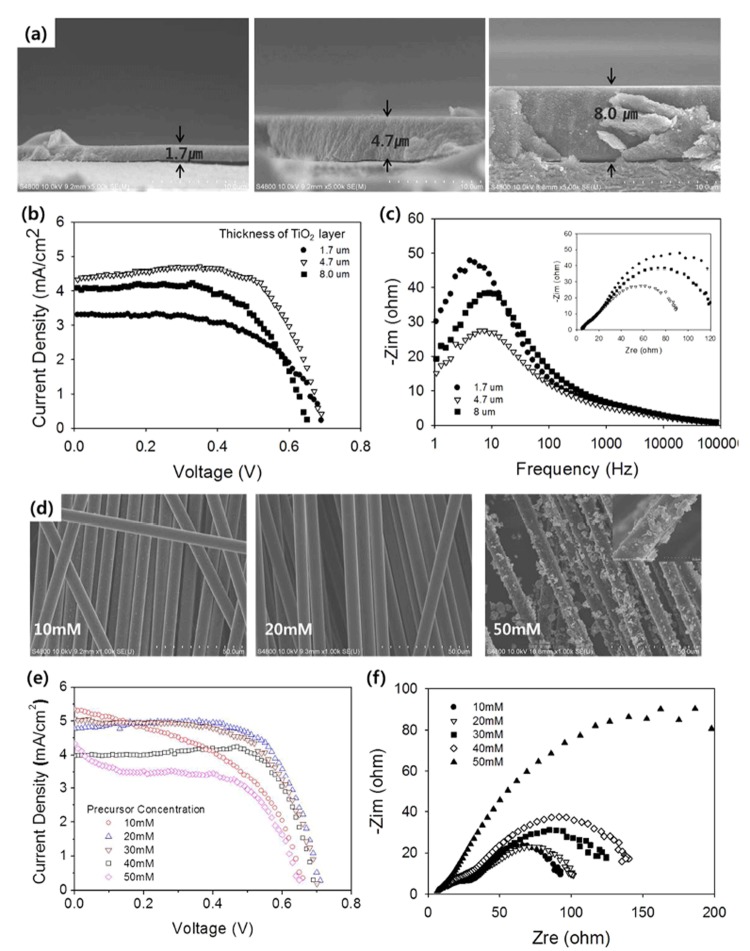
(**a**) Cross-sectional SEM images of the TiO_2_ layer deposited on holed stainless steel (SUS 304) ribbons with various thicknesses. (**b**) *J*–*V* characteristics and (**c**) electrochemical impedance spectroscopy (EIS) Bode plot from DSSCs, consisting of a photoanode shown in (**a**), a Pt-coated FTO glass as a counterelectrode, and paper as a spacer. (**d**) SEM images of Pt-loaded carbon yarn with various H_2_PtCl_6_∙6H_2_O concentrations in the dipping solution. (**e**) *J*–*V* characteristics and (**c**) Nyquist plot of inserted DSSCs in the textile with various carbon yarns as the counterelectrode. For (**e**), the TiO_2_-layer thickness was 4.7 μm. A photoanode-supporting warp diameter of 150 μm and counterelectrode-supporting warp diameter of 220 μm were used.

**Figure 4:**
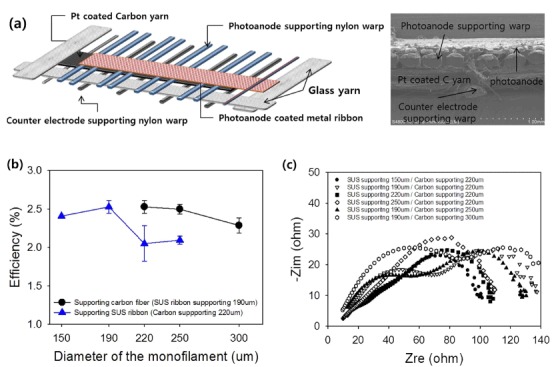
(**a**) Schematic illustration of the inserted DSSCs in the textile showing the location of the photoanode-supporting warp and counterelectrode-supporting warps. (**b**) Average energy conversion efficiency and (**c**) Nyquist plot of the inserted DSSCs in the textile with various diameters of photoanode-supporting nylon-wire warps and counterelectrode-supporting nylon-wire warps. The performances were measured using a 4.7-μm-thick TiO_2_ layer and counterelectrode carbon yarn Pt-loaded using 20 mM H_2_PtCl_6_∙6H_2_O solution by dipping and drying.

**Figure 5:**
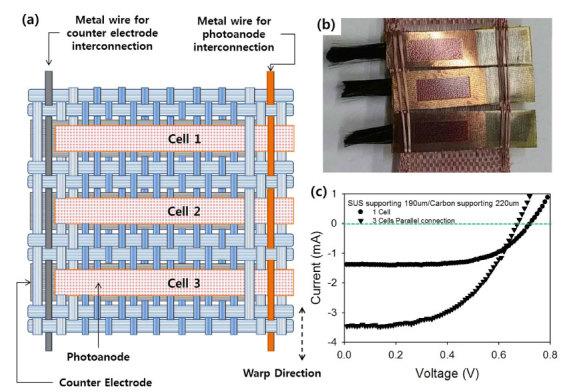
(**a**) Schematic illustration and (**b**) photograph of three DSSCs inserted in the textile and interconnected electrically by a parallel circuit with interlaying Ti wires. (**c**) Current (*I*)–voltage (*V*) characteristics of a single DSSC inserted in the textile and three parallel-interconnected DSSCs inserted in the textile.
