# Supplementary material for: Insertion of Dye-Sensitized Solar Cells in Textiles using a Conventional Weaving Process
Source: Sci Rep. 2015 Jun 18;5:11022. doi: 10.1038/srep11022 (PMC4471890; doi:10.1038/srep11022)
Supplement: Supplementary Information [file srep11022-s1.pdf]

Supplementary Information

Insertion of Dye-Sensitized Solar Cells in Textiles using a Conventional Weaving Process

Min Ju Yun[1,2], Seung I. Cha*[1], Seon Hee Seo[1], Han Seong kim[2], Dong Y. Lee[1]

1. Nano Hybrid Technology Research Center, Creative and Fundamental Research Division, Korea Electrotechnology Research Institute

2. Department of Organic Material Science and Engineering, Pusan National University

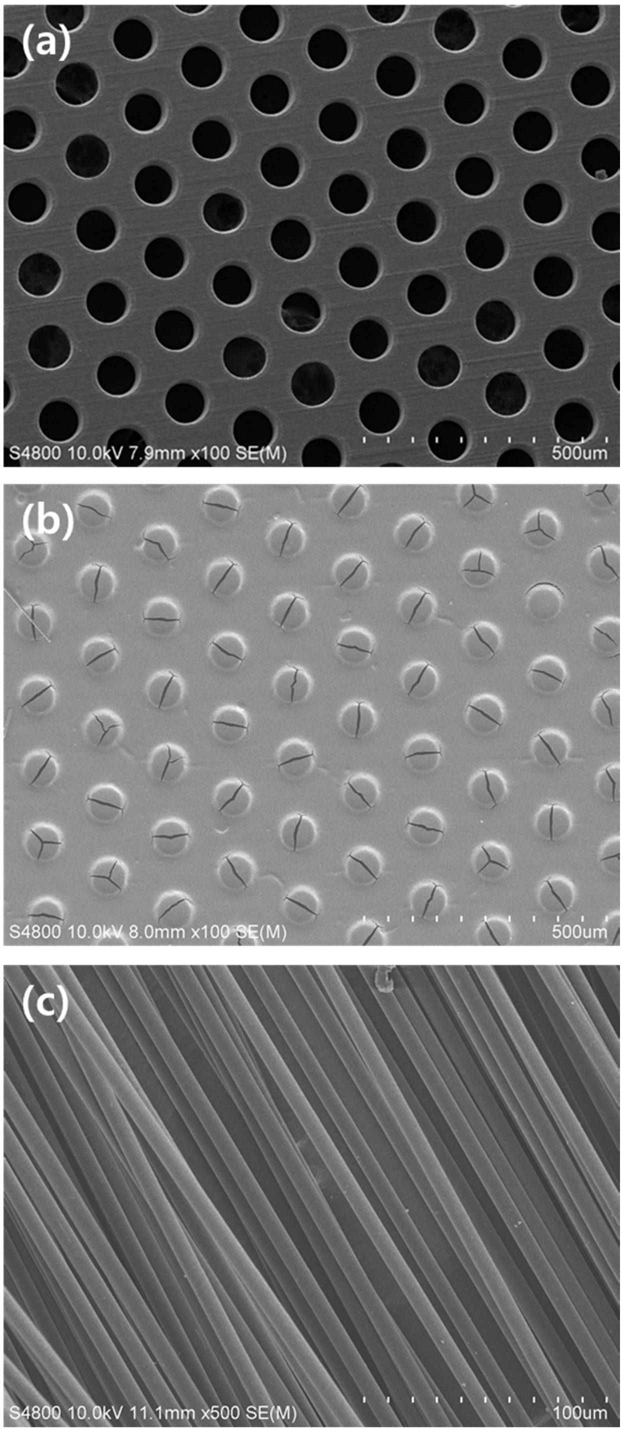

Figure S1. Scanning electron microscopy (SEM) image of (a) the etched mesh of 304 stainless steel and (b) the photoanode, (c) counterelectrode carbon yarn for the dye-sensitized solar cells (DSSCs) incorporated into the textile.

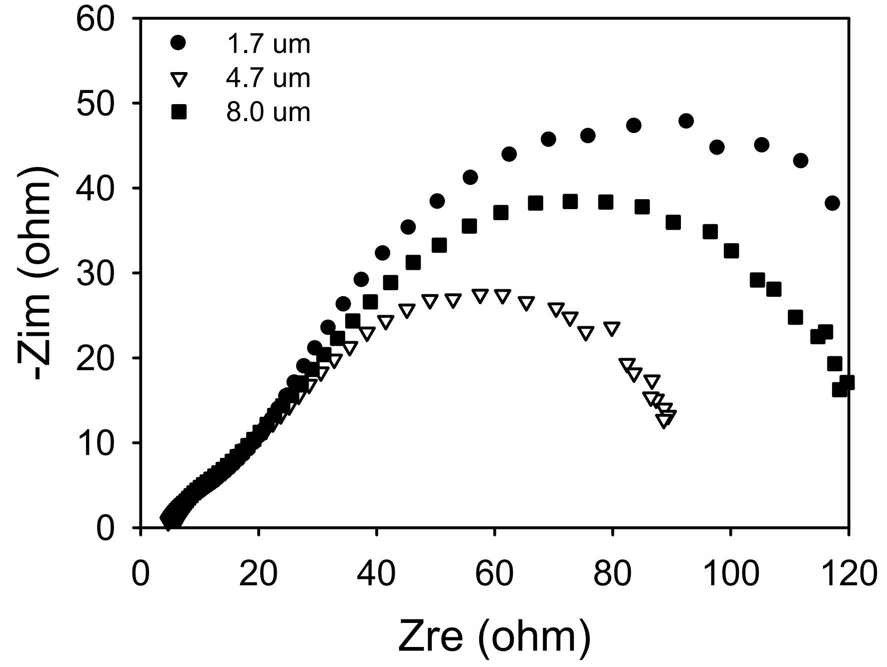

(a)

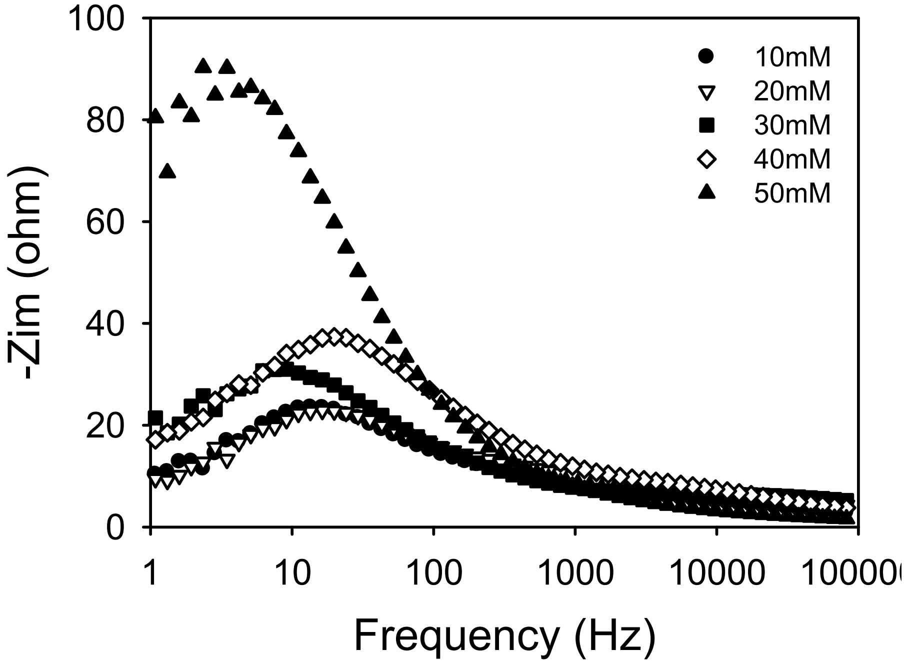

(b)

Figure S2. (a) Nyquist plot for Fig. 3(c). (b) Bode plot for Fig. 3(f).

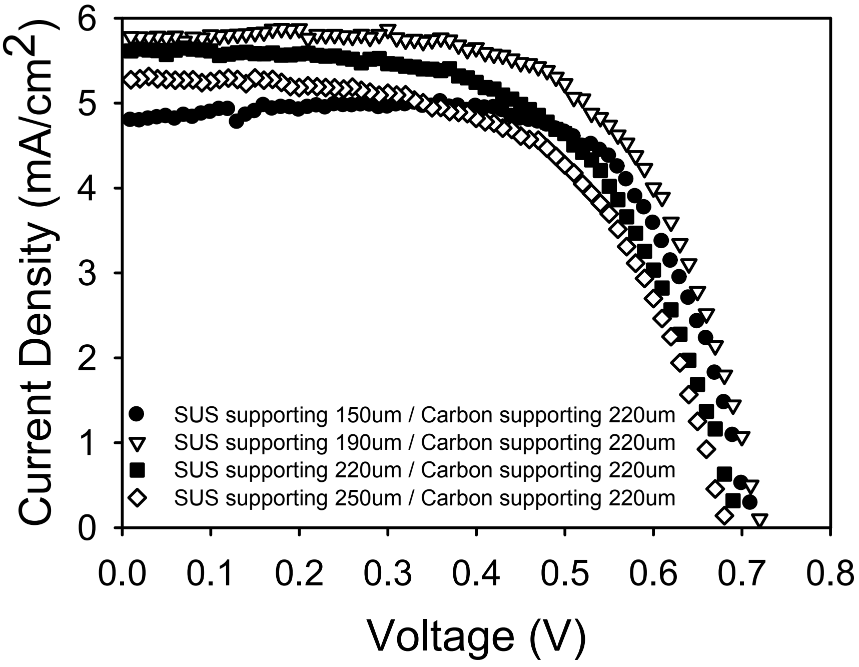

Current Density (mA/cm2)
Voltage (V)
0
1
2
3
4
5
6
0.0
0.1
0.2
0.3
0.4
0.5
0.6
0.7
0.8
SUS supporting 150um / Carbon supporting 220um
SUS supporting 190um / Carbon supporting 220um
SUS supporting 220um / Carbon supporting 220um
SUS supporting 250um / Carbon supporting 220um

(a)

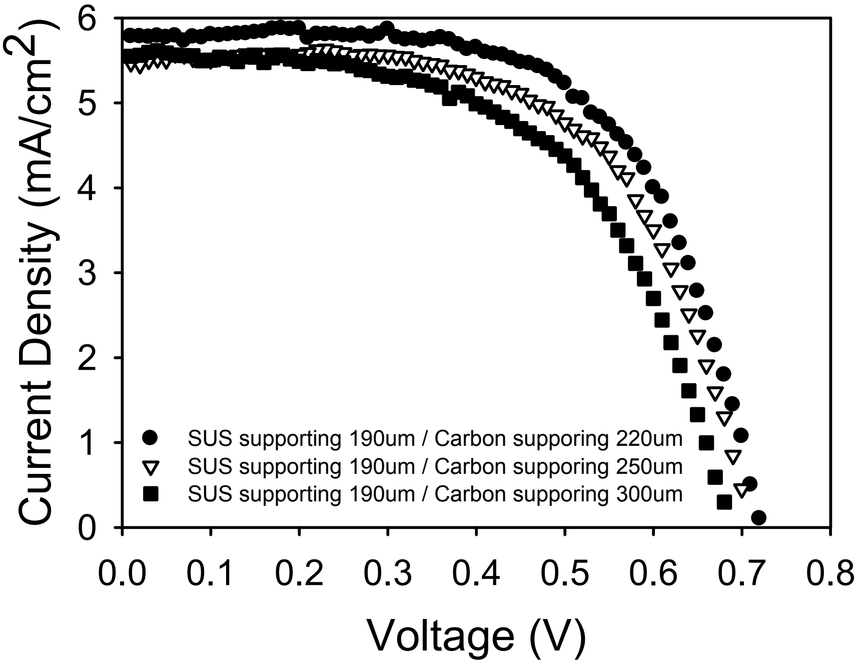

Current Density (mA/cm2)
Voltage (V)
0
1
2
3
4
5
6
0.0
0.1
0.2
0.3
0.4
0.5
0.6
0.7
0.8
SUS supporting 190um / Carbon supporing 220um
SUS supporting 190um / Carbon supporing 250um
SUS supporting 190um / Carbon supporing 300um

(b)

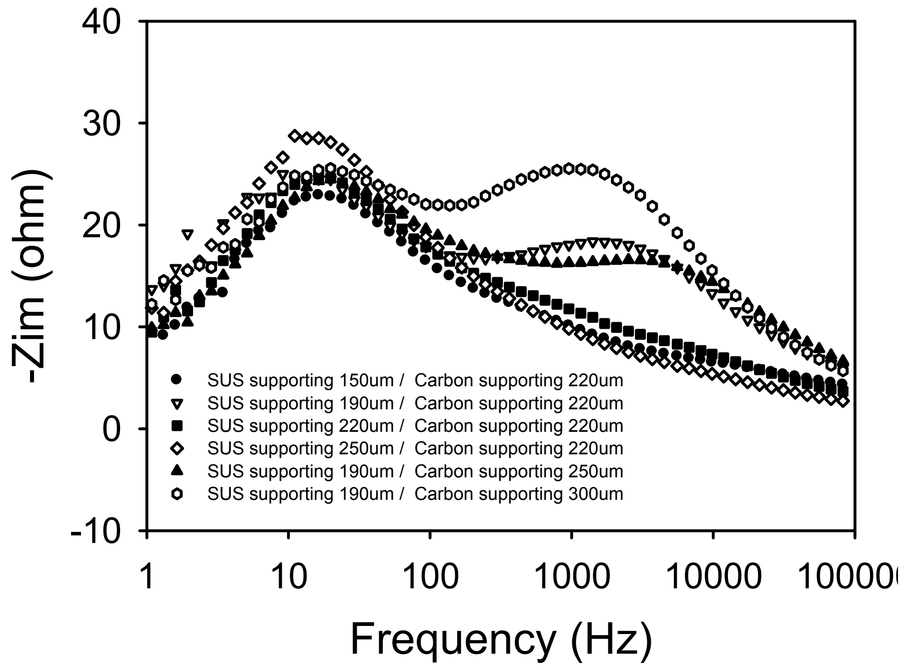

(c)

Figure S3. (a) Current density (*J*)–voltage (*V*) characteristics of DSSCs inserted in the textile with various radius of photoanode supporting nylon warps fixing the counterelectrode-supporting warp diameter of 220 μm. (b) *J*–*V* characteristics of DSSCs inserted in the textile with a various radius of counterelectrode-supporting nylon warps fixing a photoanode-supporting warp diameter of 190 μm. (c) Bode plot for Fig. 4(c).

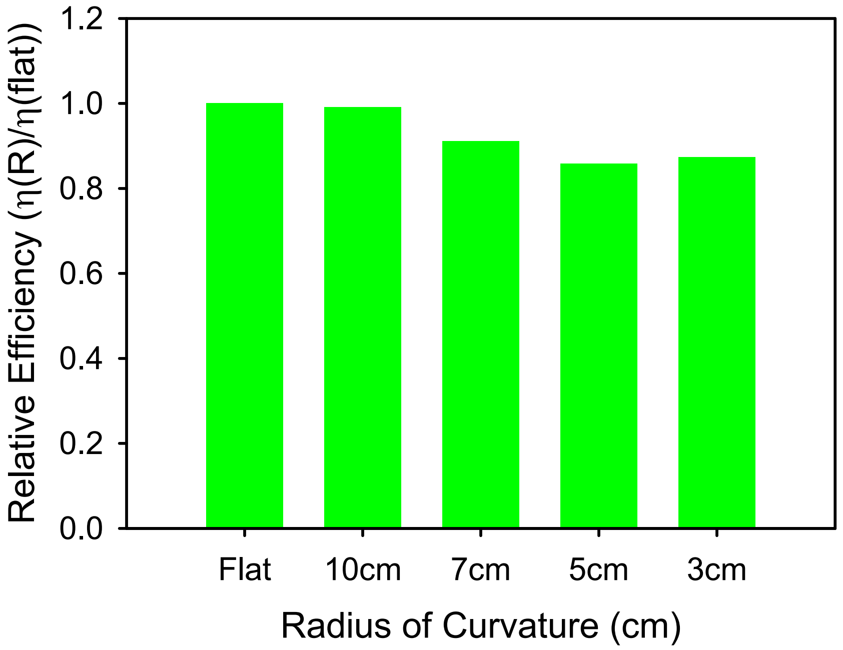

Figure S4. The relative energy conversion efficiency to flat state of inserted DSSCs according to bending radius of curvature.
